# Neuroprotective Effects of Liraglutide for Stroke Model of Rats

**DOI:** 10.3390/ijms141121513

**Published:** 2013-10-30

**Authors:** Kenichiro Sato, Masahiro Kameda, Takao Yasuhara, Takashi Agari, Tanefumi Baba, Feifei Wang, Aiko Shinko, Takaaki Wakamori, Atsuhiko Toyoshima, Hayato Takeuchi, Tatsuya Sasaki, Susumu Sasada, Akihiko Kondo, Cesario V. Borlongan, Mitsunori Matsumae, Isao Date

**Affiliations:** 1 Department of Neurological Surgery, Okayama University Graduate School of Medicine, Okayama 700-8558, Japan; E-Mails: satoken645@gmail.com (K.S.); mrkameda@gmail.com (M.K.); agarit@cc.okayama-u.ac.jp (T.A.); shinkoaiko@gmail.com (A.S.); wakagon@cc.okayama-u.ac.jp (T.W.); atsuhiko.t@kyj.biglobe.ne.jp (A.T.); tacken_osyo_fd3s_in_motion@yahoo.co.jp (H.T.); tatu_tatu_sasa@yahoo.co.jp (T.S.); sasadaminami@yahoo.co.jp (S.S.); domacv@yahoo.co.jp (A.K.); idate333@md.okayama-u.ac.jp (I.D.); 2 Department of Neurosurgery, Tokai University, Tokyo 151-0063, Japan; E-Mails: babatane@is.icc.u-tokai.ac.jp (T.B.); mike@is.icc.u-tokai.ac.jp (M.M.); 3 Center for Innovative and Translational Medicine, Kochi University Medical School, Nankoku 783-8505, Japan; E-Mail: f-wang@kochi-u.ac.jp; 4 Department of Neurosurgery, University of South Florida College Medicine, Tampa, FL 12901, USA; E-Mail: cborlong@health.usf.edu

**Keywords:** cerebral ischemia, diabetes mellitus, glucagon-like peptide-1, liraglutide, oxidative stress, stroke, VEGF

## Abstract

The number of diabetes mellitus (DM) patients is increasing, and stroke is deeply associated with DM. Recently, neuroprotective effects of glucagon-like peptide-1 (GLP-1) are reported. In this study, we explored whether liraglutide, a GLP-1 analogue exerts therapeutic effects on a rat stroke model. Wistar rats received occlusion of the middle cerebral artery for 90 min. At one hour after reperfusion, liraglutide or saline was administered intraperitoneally. Modified Bederson’s test was performed at 1 and 24 h and, subsequently, rats were euthanized for histological investigation. Peripheral blood was obtained for measurement of blood glucose level and evaluation of oxidative stress. Brain tissues were collected to evaluate the level of vascular endothelial growth factor (VEGF). The behavioral scores of liraglutide-treated rats were significantly better than those of control rats. Infarct volumes of liraglutide-treated rats at were reduced, compared with those of control rats. The level of derivatives of reactive oxygen metabolite was lower in liraglutide-treated rats. VEGF level of liraglutide-treated rats in the cortex, but not in the striatum significantly increased, compared to that of control rats. In conclusion, this is the first study to demonstrate neuroprotective effects of liraglutide on cerebral ischemia through anti-oxidative effects and VEGF upregulation.

## 1. Introduction

The number of patients with diabetes mellitus (DM) is almost nine million, and stroke is the third most common cause of death in Japan. Glucagon-like peptide-1 (GLP-1) analogue is recently established as a daily injection drug for type 2 DM, with actions including stimulation of insulin secretion, glucagon suppression, and appetite loss [1]. Liraglutide is a kind of GLP-1 derivative with a 97% homologous amino-acid sequence to GLP-1 and with addition of fatty acid [1]. A randomized multinational study demonstrated the superiority of liraglutide to exenatide, which is another GLP-1 analogue, on glycemic control with good compliance [2]. Recently, neuroprotective effects of exenatide, which has 52% homologous amino-acid sequence to GLP-1, were reported in several studies [1,3]. In this study, we investigated whether liraglutide exerted neuroprotective effects against transient cerebral ischemia induced by middle cerebral artery occlusion (MCAO).

## 2. Results

### 2.1. Behavioral Amelioration in Liraglutide-Treated Rats

**Figure 1:**
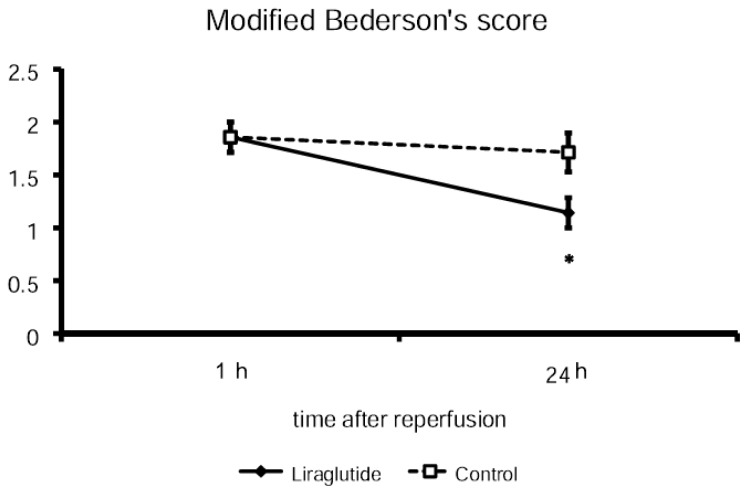
Amelioration in behavioral scores. Post-stroke administration of liraglutide reduced modified Bederson’s score at 24 h after reperfusion, although the scores of both groups did not differ at one hour after reperfusion just before administration of liraglutide or saline. * *p* < 0.05 *vs.* control group.

There was no difference in the modified Bederson’s score at one hour after reperfusion between liraglutide-treated rats (1.9 ± 0.14) and control rats (1.9 ± 0.14). At 24 h after reperfusion, the modified Bederson’s score of liraglutide-treated rats was significantly lower (1.1 ± 0.14; *p* < 0.05) than that of control rats (1.7 ± 0.18), thus indicating behavioral amelioration in liraglutide-treated rats (Figure 1).

### 2.2. Changes of Cortical Cerebral Blood Flow

**Figure 2:**
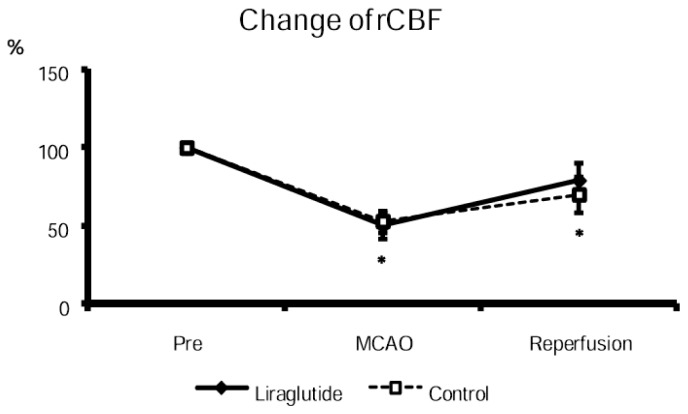
Reduced and partially recovered cortical blood flow at MCAO and reperfusion. Cortical blood flow was reduced to almost half at MCAO and recovered to 70%–80% at reperfusion. There was no difference between both groups. * *p* < 0.05 *vs.* CBF before suture insertion.

The cortical cerebral blood flow at MCAO of both groups decreased (liraglutide-treated rats: 50% ± 8.6%; control rats: 53% ± 7.1% relative to the value at pre-MCAO), compared with that at pre-MCAO. The cortical cerebral blood flow at reperfusion of both groups significantly increased, compared with that at MCAO (liraglutide-treated rats: 79% ± 11%; control rats: 70% ± 11%, Figure 2). There was no significant differences between liraglutide-treated and control rats both at MCAO and at reperfusion.

### 2.3. Effects of Liraglutide on Blood Glucose Level

Blood glucose level of liraglutide-treated rats was 98 ± 5.1 mg/dL at eight hours after reperfusion, while that of control rats was 109 ± 7.0 mg/dL. There was no significant difference between the two groups, although there seems that blood glucose level of liraglutide-treated rats might be lower than that of control rats (*p* = 0.26).

### 2.4. Reduction of Infarct Volumes in Liraglutide-Treated Rats

**Figure 3:**
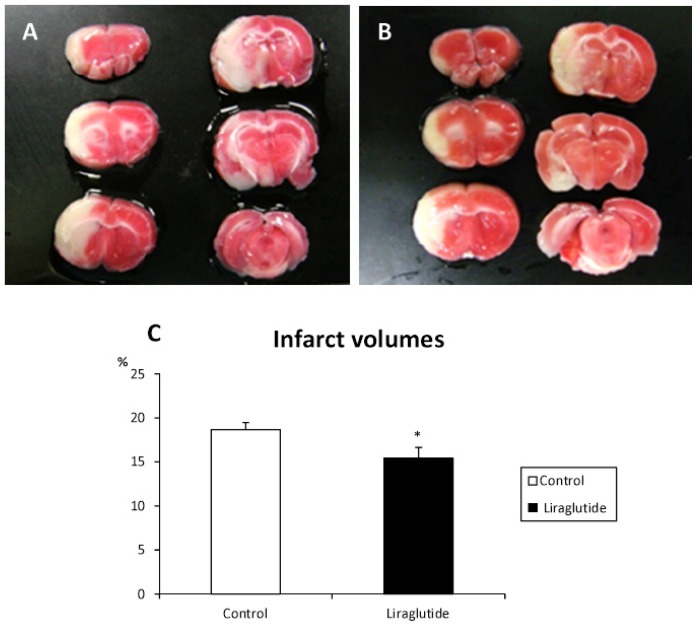
Reduced infarct volumes in rats receiving liraglutide. Representative images of TTC staining of rats in control (**A**) and liraglutide-treated groups (**B**) demonstrate remarkable reduction of infarct volumes in liraglutide-treated rats. The graph shows the significant differences in infarct volumes (**C**). * *p* < 0.05 *vs.* control group.

Representative images of 2,3,5-triphenyltetrazolium chloride (TTC) staining of liraglutide-treated and control rats are shown in Figure 3. Liraglutide-treated rats exhibited significant reduction of infarct volumes at 24 h (15.4% ± 1.3% relative to the intact side; *p* < 0.05), compared with that of control rats (18.7% ± 0.8%, Figure 3).

### 2.5. Decrease of d-ROMs in Liraglutide-Treated Rats

**Figure 4:**
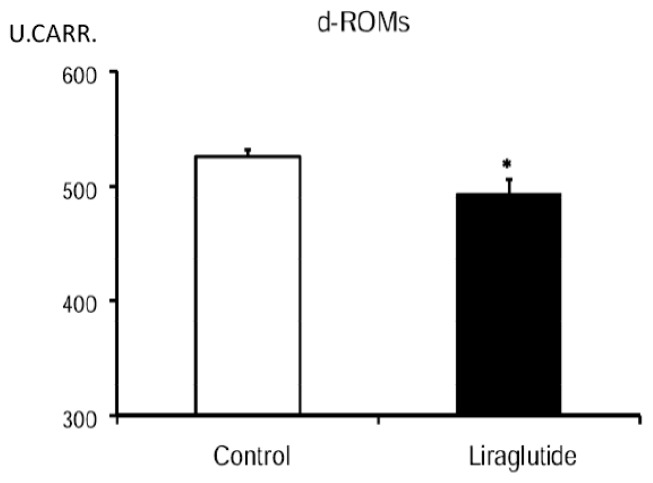
Reduced oxidative stress in liraglutide-treated rats. The graph shows significant reduction in level of d-ROMs in liraglutide-treated rats. * *p* < 0.05 *vs.* control group.

The level of derivatives of reactive oxygen metabolites (d-ROMs) in peripheral blood of liraglutide-treated rats was significantly suppressed (494 ± 12 U.CARR.) at 24 h after reperfusion, compared to that of control rats (526 ± 6 U.CARR., Figure 4).

### 2.6. VEGF Upregulation in the Cortex of Liraglutide-Treated Rats, but Not in the Striatum

**Figure 5:**
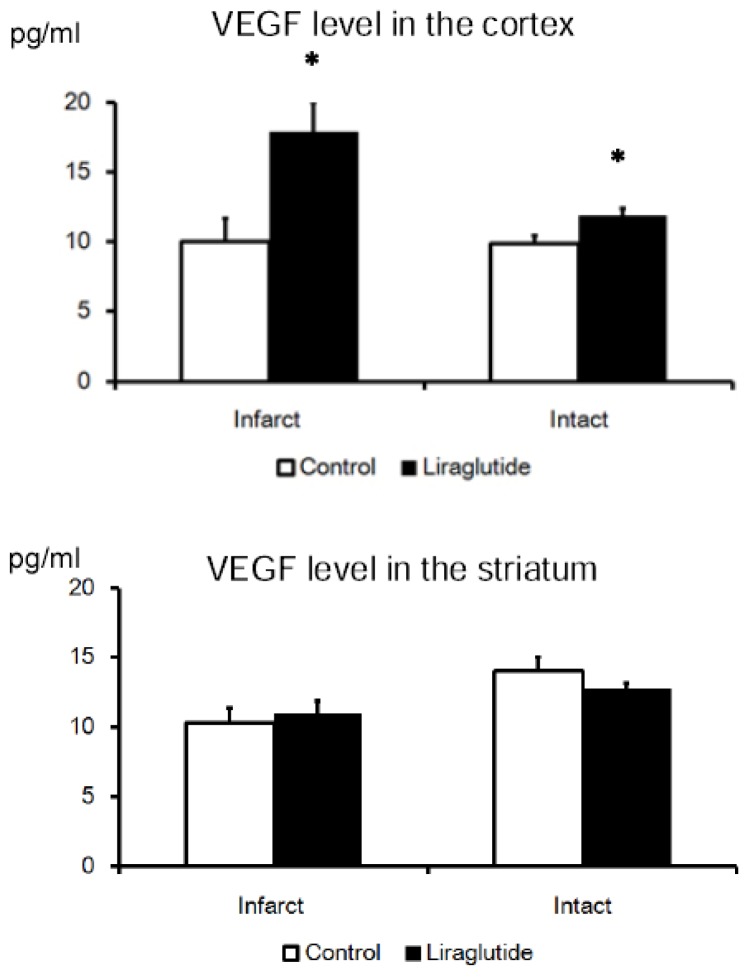
VEGF upregulation in the cortex of rats receiving liraglutide. **Upper graph**: The graph demonstrates significant increase of VEGF level in the cortex of liraglutide-treated rats; **Lower graph**: The level of VEGF in the striatum was not increased by administration of liraglutide. Infarct/Intact means tissues in the infarct/intact side, respectively. * *p* < 0.05 *vs.* control group.

Vascular endothelial growth factor (VEGF) level of the cortex in liraglutide-treated rats (infarct side: 18 ± 2.1 pg/mL, intact side: 12 ± 0.49 pg/mL) was upregulated, compared to that in rats of the control group (infarct side: 10 ± 1.7 pg/mL, intact side: 9.8 ± 0.54 pg/mL; *p* < 0.05). On the other hand, VEGF level of the striatum in liraglutide-treated rats (infarct side: 11 ± 0.88 pg/mL, intact side: 13 ± 0.48 pg/mL) was not upregulated, compared to that in rats of control group (infarct side: 10 ± 1.1 pg/mL, intact side: 14 ± 1.1 pg/mL, Figure 5).

## 3. Discussion

In this study, single administration of a GLP-1 analogue, liraglutide reduced infarct volumes induced by MCAO for 90 min with behavioral amelioration, compared to control rats. The neuroprotective effects of liraglutide might result from the suppression of oxidative stress and VEGF upregulation.

### 3.1. GLP-1 Analogue and Diseases in the Central Nervous System

Several reports demonstrated therapeutic effects of GLP-1 analogue on animal models of diseases in the central nervous system, including stroke, Parkinson’s disease and Alzheimer’s disease, and amyotrophic lateral sclerosis [4–8]. Exenatide was mainly used in those studies and neurons were protected against oxygen and glucose deprivation at least partly through the protein kinase A (PKA) pathway *in vitro* [9].

Liraglutide was a GLP-1 analogue, which has been used in several studies related to Alzheimer’s disease. Liraglutide prevented memory impairments in an Alzheimer’s disease mouse model with reduced synapse loss in the hippocampus [10]. The same group revealed that administration of liraglutide enhances long term potentiation and suppressed the deterioration of long term potentiation in Alzheimer’s disease rat model [11]. Moreover, a randomized clinical trial on Alzheimer’s disease explored whether administration of liraglutide might affect deposition of β-amyloid protein and glucose uptake in the central nervous system, cognitive function, and cerebral blood flow [12]. Recently, therapeutic effects of liraglutide on an animal model of intracerebral hemorrhage were shown to be mediated by suppression of neuroinflammation and brain edema [13]. To our knowledge, this is the first study to reveal neuroprotective effects of liraglutide on cerebral ischemia. The underlying mechanisms of neuroprotective effects by liraglutide remain to be fully understood. However, anti-oxidative effects, anti-apoptotic effects, neurogeneic effects, and neurotrophic effects might have contributed to the observed therapeutic effects of liraglutide.

### 3.2. Anti-Oxidative Effects of GLP-1 and Its Analogue

In our study, the level of d-ROMs of liraglutide-treated rats significantly reduced, compared with that of control rats. The mechanisms of the reduced level of d-ROMs in blood may be due to the suppressed oxidative stress in the ischemic brain produced by liraglutide administration. There has been no study directly demonstrating anti-oxidative effects of liraglutide in the central nervous system. Previously, a few reports demonstrated anti-oxidative effects of exenatide and GLP-1 on pancreatic islet cells [14,15]. Administration of GLP-1 suppressed expression of inducible nitric oxide synthase (iNOS) in pancreatic islet cells induced by glucose at least partly through PKA signaling [15]. In another study, exenatide exerted therapeutic effects after islet transplantation in mice through anti-oxidative effects and anti-apoptotic effects [14]. Exenatide also showed anti-oxidative effects on endothelial cells through normalizing endothelial NOS (eNOS) upregulation with subsequent release of reactive oxygen species. These findings advance the key role of anti-oxidative signaling pathways in exenatide’s therapeutic benefits [16]. Indeed, exenatide showed neuroprotective effects on Parkinson’s disease mouse model, induced by 1-methyl-4-phenyl-1,2,3,6-tetrahydropyridine (MPTP), which might also suggest that therapeutic effects of exenatide were strongly related to anti-oxidative effects [8].

### 3.3. Other Neuroprotective Mechanisms of GLP-1 Including Secretion of Neurohumoral Factors

In ischemic brain, neurogenesis increased with migration of newly developed progenitor cells into ischemic penumbra. Enhancement of endogenous neurogenesis might be a promising strategy of regenerative medicine for diseases in the central nervous system [17]. As described above, liraglutide was used for research in Alzheimer’s disease. In several studies, neurogeneic effects of liraglutide were shown, in that the administration of liraglutide, as well as exenatide, resulted in enhanced proliferation of progenitor cells with a subsequent increase in young neurons in the dentate gyrus of high-fat-diet-fed mice [18] and Alzheimer’s disease mouse model [10]. It was also demonstrated in another study that doublecortin-positive neural progenitor cells and NeuN-positive mature neurons increased in the hippocampus [19]. Furthermore, lixisenatide, a newly developed GLP-1 analogue exerted stronger neurogeneic effects than liraglutide on Alzheimer’s disease rodent model, although both agents breached the blood brain barrier and were bioavailable in the brain with subsequent neurogeneic effects [20].

Recent studies revealed that GLP-1 enhances proliferation and differentiation of endothelial progenitor cells through upregulation of vascular endothelial growth factor [21]. Growth factors or trophic factors upregulated by GLP-1 analogue might have contributed to the therapeutic effects seen in this study. In our study, VEGF level in the cortex, but not in the striatum, of liraglutide-treated rats was upregulated, compared to that of rats in the control group. Moreover, anti-inflammatory effects of liraglutide might have also influenced the results of this study, as shown in previous Alzheimer’s disease research [10]. Of note, VEGF exerts neuroprotective, angiogeneic, neurogeneic, and anti-inflammatory effects on various diseases of the central nervous system [22,23]. Further studies are needed to explore the expression of other neurotrophic factors to fully recognize the therapeutic potential of liraglutide, in view of accumulating evidence that neurohumoral factors secreted from various cells are key to the neuroprotective and neurorestorative effects of cell-based therapies for brain disorders [24].

## 4. Experimental Section

### 4.1. Subjects

Adult male Wistar rats (CLEA JAPAN, Inc., Tokyo, Japan; *n* = 49) weighing 200 to 250 g at the beginning of the experiment, according to approved guidelines of the Institutional Animal Care and Use Committee of Okayama University. Three rats were excluded in this study for severe subarachnoid hemorrhage and two for failure of model making with completely preserved motor function. They were singly housed per cage in a temperature- and humidity-controlled room, maintained on a 12-h light/dark cycle, with free access to food and water.

### 4.2. Experimental Groups

**Figure 6:**
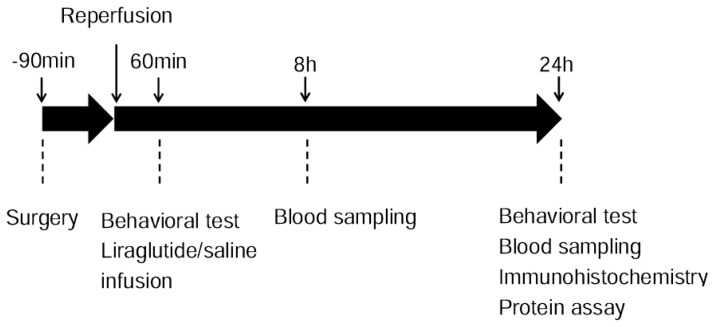
Experimental design. Rats in control and liraglutide-treated groups underwent MCAO surgery with subsequent reperfusion at 90 min after the occlusion. Behavioral test was performed at 1 and 24 h after reperfusion. Blood was sampled from caudal vein to evaluate oxidative stress and blood glucose level. Then rats were euthanized for histological evaluation and protein assay at 24 h.

Rats were randomly divided into two groups, that is, control (*n* = 22) or liraglutide-treated group (*n* = 22). At one hour after reperfusion, either normal saline (1 mL, to control rats) or liraglutide (1 mL, 700 μg/kg, to liraglutide-treated rats, Novo Nordisk Pharma Ltd., Gladsaxe, Denmark) was administered intraperitoneally. The dose of liraglutide and timing of administration used in our study was determined by previous studies on DM [25] and our preliminary study (data not shown). At 24 h after reperfusion, behavioral tests were performed with subsequent euthanasia for histological analyses or protein assay (Figure 6).

### 4.3. Transient Middle Cerebral Artery Occlusion

MCAO was carried out according to the intraluminal suture method used in our previous study [26,27]. In brief, all rats were anesthetized with 1% halothane in 69% N_2_O and 30% O_2_. A 4-0 monofilament nylon suture with silicone-coated tip (Xantopren L blue & ACTIVATOR Universal Liquid, Heraeus Kulzer GmbH & Co. KG, Hanau, Germany) was inserted through an arteriotomy in the right common carotid artery. The middle cerebral artery was occluded for 90 min, whereas the rats were allowed to move freely after awakening in a cage. Consequently the rats were re-anesthetized and the middle cerebral artery was recanalized.

### 4.4. Measurement of Cortical Cerebral Blood Flow

Cortical cerebral blood flow at the right parietal cortex was measured on MCAO after suture insertion and reperfusion immediately after recanalization by using a laser Doppler device (Laser flowmeter ALF21; Advance Company, Ltd., Saitama, Japan). The stainless steel probe (diameter: 6 mm) was placed on the brain surface through the hole perforated on the right parietal bone.

### 4.5. Neurological Evaluation: Modified Bederson’s Score

Neurological deficits were evaluated using modified Bederson’s score at one hour after reperfusion, immediately before liraglutide-administration and 24 h after reperfusion (*n* = 7 in each group). The grades were defined as follows: grade 0: no observable deficit; grade 1: left forelimb flexion with the extension of the other forelimb straight when the rat was suspended by 20–30 cm above the testing table; grade 2: decreased resistance to lateral push (and forelimb flexion) without circling; grade 3: same behavior as grade 2, with circling [28,29].

### 4.6. Measurement of Blood Glucose Level

Peripheral blood was obtained from caudal vein at eight hours after reperfusion. Blood glucose was measured by using a blood glucose meter, ONETOUCH Ultra Vue (Johnson & Johnson Corp., New Brunswick, NJ, USA).

### 4.7. Measurement of the Infarct Volumes

Rats were euthanized under deep anesthesia using pentobarbital (100 mg/kg) with following saline perfusion and brains were quickly removed at 24 h after reperfusion for TTC staining (*n* = 7 in each group) [26]. Thereafter, six serial coronal sections of 2-mm thickness were prepared. Brain slices were incubated in a 0.2% solution of TTC (Kanto Chemistry Co., Tokyo, Japan) in phosphate buffered saline (PBS) at 37 °C for 30 min and fixed by immersion in 4% buffered formaldehyde solution. The normal area of brain was stained dark red based on intact mitochondrial function, whereas infarct area remained unstained. Each brain slice was scanned by a color flatbed scanner, and infarct area was measured using a computerized image analysis using Image J software. Infarct volumes were calculated as described previously [26,27] and the ratio to the whole brain was statistically analyzed.

### 4.8. Measurement of Oxidative Stress

The blood was taken from caudal vein by cutting at 24 h after reperfusion and the level of oxidative stress was measured (*n* = 7 in each group). The level of d-ROMs in blood was measured by a free radical elective evaluator: FREE carpe diem (Wismerll Company Ltd., Tokyo, Japan) according to the analytical manual. The results of the d-ROMs test was expressed as U. CARR (1 U.CARR. corresponds to 0.08 mg/dL of H_2_O_2_) [30].

### 4.9. VEGF ELISA

For protein assay, fresh brain tissues of the bilateral cortex and striatum from rats of control and liraglutide-treated groups (*n* = 8 in each group) were quickly removed after decapitation using overdosed pentobarbital (100 mg/kg, i.p.) at 24 h after reperfusion. Brains were sliced at a thickness of 2 mm. The brain tissues of the cortex and striatum were punched out using a biopsy punch (2 mm-hole, Kai corporation and Kai industries co., ltd, Tokyo, Japan). Brain tissues were then homogenized in T-PER (Pierce, Rockfold, IL, USA) and centrifuged at 10,000 × *g* for 10 min at 4 °C, and the supernatant was obtained. Tissue VEGF level was measured by the usage of rat VEGF ELISA assay kit (IBL, Gunma, Japan).

### 4.10. Statistical Analyses

Data are presented as the mean ± S.E. (modified Bederson’s score; infarct volumes: percent to the intact hemisphere; d-ROM: Carr U; VEGF: pg/mL). Data were evaluated statistically using Mann–Whitney *U* test. Data of VEGF level were evaluated using repeated-measures ANOVA or one-way ANOVA, followed by *post hoc* Scheffe’s test. Statistical significance was preset at *p* < 0.05.

## 5. Conclusions

In this study, reduction of infarct volumes with behavioral improvement was achieved by administration of liraglutide likely through anti-oxidative effects and VEGF upregulation. Liraglutide may afford therapeutic benefits to type 2 DM patients with cerebral infarcts, although further basic and clinical research investigations are needed.
